# Supplementary figures and images for: NODeJ: an ImageJ plugin for 3D segmentation of nuclear objects
Source: BMC Bioinformatics. 2022 Jun 6;23:216. doi: 10.1186/s12859-022-04743-6 (PMC9169307; doi:10.1186/s12859-022-04743-6)

A

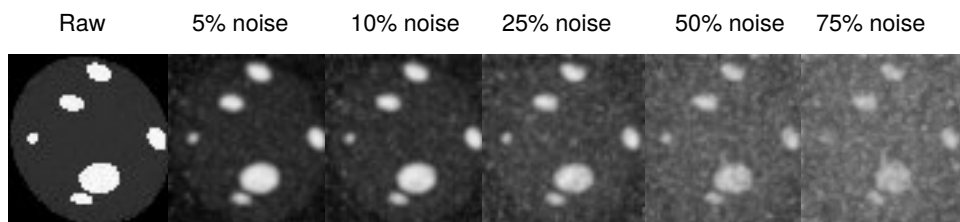

B

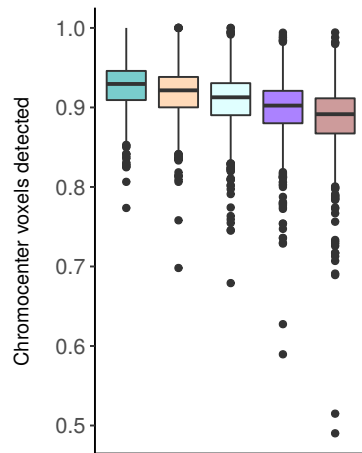

C

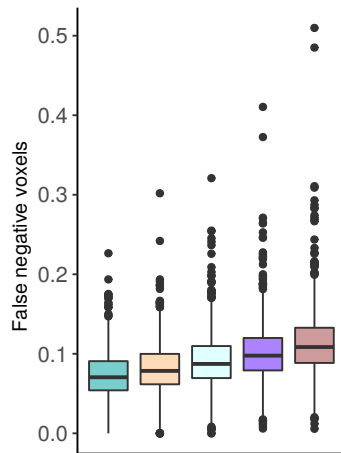

D

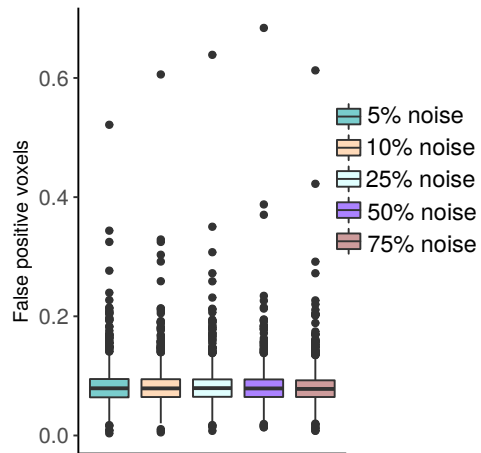

E

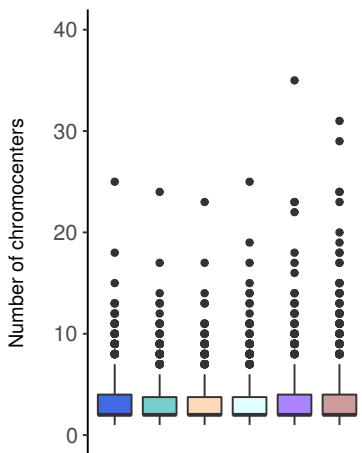

F

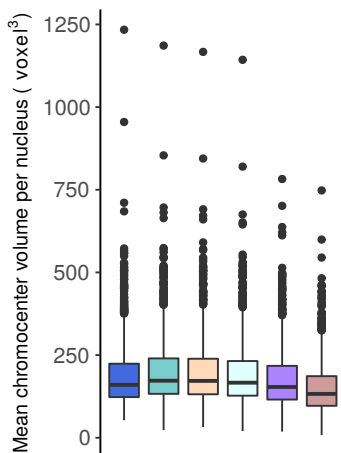

G

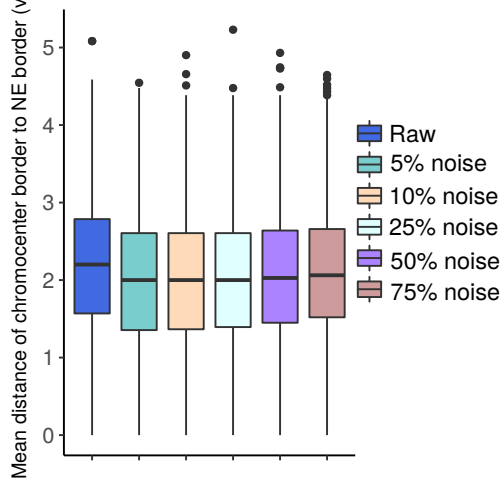

Supplement: Supplementary file 4 — Additional file 4: Fig. S1. Robustness of NODeJ object detection in relation to image noise. Briefly,an artificial dataset of 1526 individual 3D images of nuclei was generated, and different levels of noise were added to generate 9156 images. All images showed variation in the number, position and volume of the chromocenters. We used 3DTools (https://framagit.org/mcib3d/mcib3d-core/-/wikis/home) to make ellipsoids from our initial binary dataset and then shifted the position of chromocenters inside the nucleus [23]. Increasing percentages of salt and pepper noise (5%, 10%, 25%, 50% and 75%) were added with the ImageJ method [14]. NODeJ was then used to calculate the chromocenter parameters within the images using the parameter -isG (Additional file 1). A. Image representing the z-projection of an artificial nucleus with increasing levels of noise. B. Fraction of common voxels between the raw images and images with noise (true positive signal). C. The voxels missed by NODeJ due to the noise (i.e. false negative signal). D. False positive voxels, which are signals detected by NODeJ only on images with noise. E. The number of chromocenters. F. Mean chromocenter volume per nucleus. G. Mean distance from the chromocenter border to the nuclear envelope per nucleus. The box plots were made using various R packages [20, 21] (Additional file 2 and 3 describe the computed parameters) [file 12859_2022_4743_MOESM4_ESM.pdf]

A

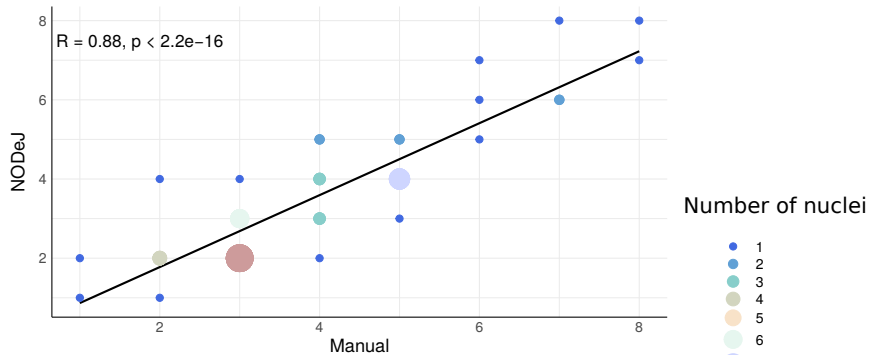

B

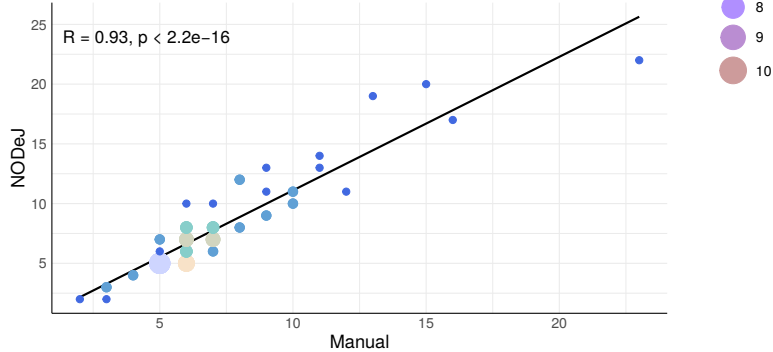

Supplement: Supplementary file 5 — Additional file 5: Fig. S2. Comparison of results obtained by manual count or with NODeJ. A.Number of chromocenters per nucleus detected manually or by using NODeJ. We manually counted the chromocenters of 52 nucleus images and compared those results with results obtained from NODeJ on the same images. The Pearson correlation factor (R) shows a significant correlation between these two results. B. Number of 180bp FISH signals per nucleus detected manually or via NODeJ. We manually counted signals from 61 nucleus images and compared the results with FISH signals detected using NODeJ. Scatter plots and statistical tests were made using various R packages [20, 21] (Additional file 2 and 3 describe the computed parameters). [file 12859_2022_4743_MOESM5_ESM.pdf]
