# Supplementary material for: NODeJ: an ImageJ plugin for 3D segmentation of nuclear objects
Source: BMC Bioinformatics. 2022 Jun 6;23:216. doi: 10.1186/s12859-022-04743-6 (PMC9169307; doi:10.1186/s12859-022-04743-6)
Supplement: Supplementary file 3 [file 12859_2022_4743_MOESM2_ESM.docx]

NucleusFileName	Nucleus image file name
Volume	Volume of segmented object (sum of white voxels)
Flatness	Computed by ratio between major and minor axes of the segmented object
Elongation	Computed by ratio between major and medium axes of the object
Sphericity	Computed by ratio between surface area and volume
Esr	Equivalent spherical radius, estimated radius computed from the volume
SurfaceArea	Segmented object surface area
MeanIntensityNucleus	Mean voxels intensity of segmented image
MeanIntensityBackground	Mean intensity of the background
StandardDeviation	Standard deviation of the segmented object intensity
MinIntensity	Minimum voxel intensity of the segmented object
MaxIntensity	Maximum voxel intensity of the segmented object
MedianIntensityImage	Median voxel intensity of raw image
MedianIntensityNucleus	Median voxel intensity of segmented object
MedianIntensityBackground	Median voxel intensity of background
ImageSize	Number of voxel
VolumeRHF	Volume of Relative Heterochromatin Fraction
NbCc	Number of chromocenters
VCcMean	Mean chromocenters volume
VCcTotal	Total chromocenters volume
DistanceBorderToBorderMean	Mean distance of chromocenters border to nuclear periphery
DistanceBarycenterToBorderMean	Mean distance of chromocenters barycenter to nuclear periphery

NucleusFileName	Nucleus image file name
Area	Nucleus mask area
Perimeter	Nucleus mask perimeter
Circ	Nucleus mask circularity
AR	Nucleus mask aspect ratio
Solidity	Nucleus mask solidity
Round	Nucleus mask roundness
NbCC	Number of chromocenters
RHF	Relative Heterochromatin Fraction
AreaCc	Chromocenter area
