# Supplementary material for: NODeJ: an ImageJ plugin for 3D segmentation of nuclear objects
Source: BMC Bioinformatics. 2022 Jun 6;23:216. doi: 10.1186/s12859-022-04743-6 (PMC9169307; doi:10.1186/s12859-022-04743-6)
Supplement: Supplementary file 4 [file 12859_2022_4743_MOESM3_ESM.doc]

NucleusFileName	Nucleus image file name
Volume	Chromocenter volume
BorderToBorderDistance	Distance of chromocenters border to nuclear periphery
BarycenterToBorderDistance	Distance of chromocenters barycenter to nuclear periphery
